# Posterior segment complications of Ahmed valve implantation

**DOI:** 10.1186/s12886-022-02297-y

**Published:** 2022-02-15

**Authors:** Yuli Park, Kyong Jin Cho

**Affiliations:** Department of Ophthalmology, Dankook University Hospital, College of Medicine, Dankook University, Cheonan, Republic of Korea

**Keywords:** Ahmed valve implantation, Posterior segment complication

## Abstract

**Background:**

To assess the time, incidence, and outcome of posterior segment complications of Ahmed valve implantation (AVI).

**Methods:**

248 eyes that underwent AVI were reviewed retrospectively. Visual acuity, preoperative characteristics, and postoperative posterior segment complications were assessed.

**Results:**

The incidence of posterior segment complications of AVI was 31.4% (78/248). The mean follow-up period was 97.4 ± 53.5 months. The mean time to occur posterior segment complication was 1.5 months. The most common posterior segment complication was choroidal detachment (17.7%) and others included ocular decompression retinopathy (3.2%), hypotonic maculopathy (2.8%), vitreous hemorrhage (2.0%), retinal detachment (1.2%), endophthalmitis (1.2%), suprachoroidal hemorrhage (1.2%), epiretinal membrane (0.8%), cystoid macular edema (0.8%), and proliferative vitreoretinopathy (0.4%). Older age, hypertension, and postoperative hypotony had an increased risk of posterior segment complications of AVI.

**Conclusions:**

The overall incidence of posterior segment complications of AVI was 31.4%. Older age, hypertension, and postoperative hypotony were significantly associated with posterior segment complications of AVI.

## Background

The purpose of the glaucoma surgery is to decrease intraocular pressure (IOP) by producing a new aqueous humor outflow pathway, and hence avoid further damage of optic nerve. In contrast to trabeculectomy, Ahmed valve implantation (AVI) yields a different drainage pathway of aqueous humor bypassing dysfunctional anterior chamber angle [1]. It has been a vital technique for refractory glaucoma after multiple failed surgeries. The aqueous shunt has been used increasingly in the treatment of medically uncontrolled glaucoma and it is becoming more common to utilize glaucoma drainage implants for refractory glaucoma [2]. In 2008 American Glaucoma Society survey there was a important alteration in treatment pattern, showing that the use of trabeculectomy was decreased from 81 to 46% and the use of glaucoma drainage device was increased from 18 to 51% [3]. The stimulus for the diversion could be grounded on the outcomes of the Tube Versus Trabeculectomy (TVT) study [4, 5]. However, the elucidation of the outcome is confounded by the intricacy of study design [6].

The aim of our study is to describe time, incidence, and outcomes of posterior segment complications of AVI, so as to allow relevant management to be conducted after AVI.

## Methods

Our study adhered to the tenets of the Declaration of Helsinki and was approved by the Institutional Review Board of Dankook university hospital (IRB#DKUH202010033). The medical records of patients who were treated with AVI to control intractable IOP elevation from January, 2004 through August, 2020 were reviewed retrospectively. Patients with post-operative follow-up period > 36 months, IOP remained higher than 21 mmHg even after using glaucoma medications, and without mental illness were included. Only one eye was recruited from a single patient. Patients with concomitant treatments, such as silicone oil removal, penetrating keratoplasty, or cataract extraction during AVI were excluded.

Data were collected before and after AVI including known medical and ocular history, age, previous posterior segment disease, indication for surgery, number of AVI, best corrected visual acuity (BCVA), IOP, postoperative posterior segment complications, and medical or surgical interventions during the follow-up time. The patients were evaluated preoperatively, at postoperative 1 day, 1, 2 week, 1, 2, 3, 6 months, and thereafter yearly and were assessed by slit-lamp biomicroscopy, gonioscopy, funduscopy, Goldmann applanation tonometry at each visit. Standard automated perimetry (Humphrey Visual Field Analyzer; Carl Zeiss-Meditec Inc., Dublin, CA, USA), optical coherence tomography (OCT, Carl zeiss, Jena, Germany), and color disc photography (FF450 with Visupac, Carl Zeiss Meditec) were used to evaluate glaucoma control. IOP reading was examined 3 times to calculate the average value. Visual acuity (VA) was measured with Hahn’s visual acuity chart (Hahn’s Co. Ltd., Seoul, Korea) placed 5 m from the subject. The original data for BCVA was collected in Snellen values and converted to LogMAR for statistical analysis. Postoperative posterior segment complications of AVI were defined as any pathologic lesion which was not present before AVI located on the posterior segment and diagnosed with clinical fundus examination, B scan ultrasonography, or OCT.

### Ahmed valve implantation

**Fig. 1:**
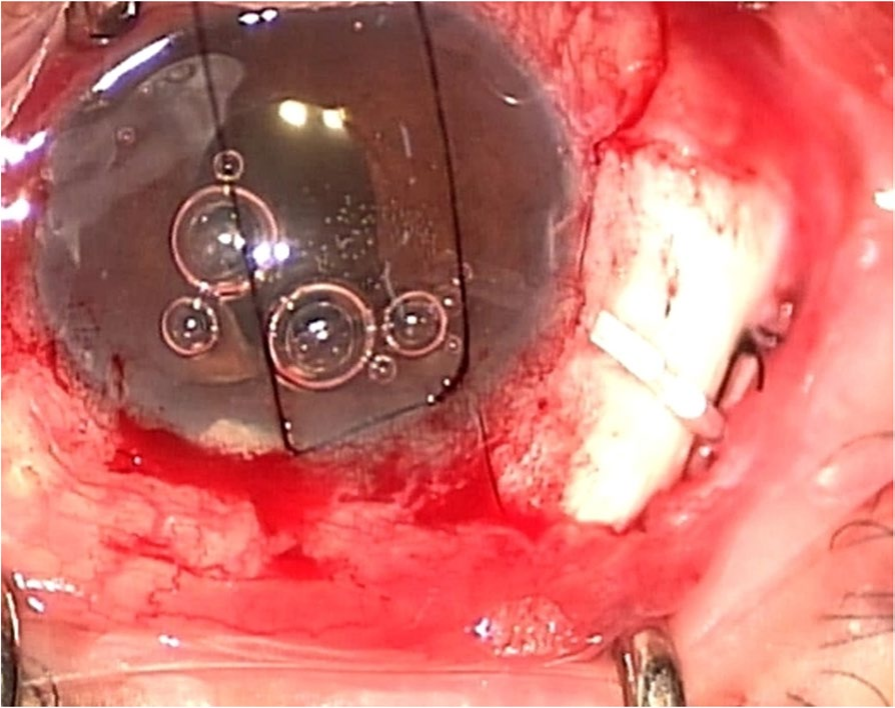
Intraoperative image of Ahmed valve implantation

Ahmed valves model FP7 (New World Medical, Rancho Cucamonga, California, USA) were used during all operations. Fornix-based incision was created through tenon capsule and conjunctiva. After priming the valve, the body was inserted and secured to the sclera 8 mm posteriorly from corneoscleral limbus (Fig. 1). The tube was cut and placed into anterior chamber. A sclera patch was sutured to cover the valve on sclerostomy site and the tenon and conjunctiva was approximated. Topical antibiotics and steroids were used after AVI.

### Statistical analysis

The categorical variables were compared by a chi square test. t test was used to compare the data of patients with posterior segment complications and those without. The factors associated with the posterior segment complications of AVI were evaluated by multivariate Cox proportional hazard regression. SPSS statistical software (version 19.0; SPSS Science Inc., Chicago, IL, USA) was used and *p* values less than 0.05 were deemed statistically significant.

## Results

248 eyes of 248 patients who underwent AVI were included. The patients were 132 (53.2%) men and 116 (46.8%) women, and the mean age was 65.7 ± 11.6 years. The mean follow-up time was 97.4 ± 53.5 months. The indications for AVI were 102 (41.1%) cases of neovascular glaucoma, 51 (20.6%) cases of uveitic glaucoma, 23 (9.3%) cases of pseudoexfoliative glaucoma, 40 (16.1%) cases of primary open angle glaucoma, and 32 (12.9%) cases of other secondary glaucoma (Table 1).

**Table 1:** Demographic characteristics of patients who underwent Ahmed valve implantation

Characteristics	
Eyes (n)	248
Male: Female (n)	132: 116
Age, mean ± SD (years)	65.7 ± 11.6
Lens status (n)	
Phakic	117
Aphakic	6
Pseudophakic	125
Medical history (n)	
Diabetes	96
Hypertension	99
Preoperative IOP (mmHg)	38.6 ± 12.1
History of cataract surgery (%)	46.4
History of glaucoma surgery (%)	28.6
Preoperative diagnosis (n)	
Neovascular glaucoma	102
Uveitic glaucoma	51
Primary open angle glaucoma	40
Pseudoexfoliative glaucoma	23
Other secondary glaucoma	32

78 eyes (31.4%) showed posterior segment complication after AVI. The mean time of occurring posterior segment complications was 1.5 months. Table 2 shows BCVA and IOP between eyes with posterior segment complications and those without. Before AVI, no significant difference was shown in the mean preoperative BCVA (logMAR ± SD: 0.7 ± 0.4, 0.6 ± 0.3, respectively, *p* = 0.61), but, the mean final postoperative BCVA was significantly worse in eyes with posterior segment complications compared to the eyes without (logMAR ± SD: 1.4 ± 0.5, 0.7 ± 0.3, respectively *p* = 0.004). In 78 eyes with posterior segment complications, 85.8% had postoperative hypotony (IOP < 6 mmHg) on one or more postoperative evaluations for 2 to 35 days. Patients presenting with hypertension had an elevated hazard ratio (HR) of 1.63 (95% confidence interval [CI] 1.05–2.73, *p* = 0.004). There was an increased risk of posterior segment complications for patients with postoperative hypotony (HR 6.52, 95% CI 1.83–21.45, *p* = 0.002). Older age had an elevated HR of 1.35 (95% CI 0.97–2.05, *p* = 0.004). Patients presenting with diabetes had HR of 1.27 (95% CI 0.69–1.93, *p* = 0.076). Higher mean preoperative IOP had HR of 1.14 (95% CI 0.53–2.16, *p* = 0.083).

**Table 2:** Comparison between eyes with and without posterior segment complications of Ahmed valve implantation

	Eyes with posterior segment complication	Eyes without posterior segment complication	*P*
Male (%)	60.2	51.1	0.34**
Medical History Diabetes mellitus (n)	44	52	0.49**
Hypertension (n)	71	28	0.03**
Age (years)	74.3 ± 15.6	62.8 ± 13.4	0.01*
Mean preoperative BCVA (LogMAR±SD, Snellen equivalent)	0.7 ± 0.4 (20/102)	0.6 ± 0.3 (20/89)	0.61*
Mean final postoperative BCVA (LogMAR±SD, Snellen equivalent)	1.4 ± 0.5 (20/537)	0.7 ± 0.3 (20/115)	0.004*
Mean preoperative IOP (mmHg)	37.4 ± 11.8	39.7 ± 12.4	0.73*
Mean final postoperative IOP (mmHg)	15.5 ± 4.7	16.2 ± 3.4	0.58*
Postoperative hypotony (IOP < 6 mmHg) (%)	85.8	32.4	0.001**

* Independent samples t test was used

**Chi-square test was used

The incidence of posterior segment complications of AVI is summarized in Table 3. The most common posterior segment complication was choroidal detachment (44 eyes; 17.7%), followed by ocular decompression retinopathy (8 eyes; 3.2%), hypotonic maculopathy (7 eyes; 2.8%), vitreous hemorrhage (5 eyes; 2.0%), endophthalmitis (3 eyes; 1.2%), retinal detachment (3 eyes; 1.2%), suprachoroidal hemorrhage (3 eyes; 1.2%), cystoid macular edema (2 eyes; 0.8%), epiretinal membrane (2 eyes; 0.8%), and proliferative vitreoretinopathy (1 eye; 0.4%). The majority of choroidal detachment, ocular decompression retinopathy, and suprachoroidal hemorrhage developed within postoperative 1 month, but most cases of epiretinal membrane and retinal detachment were revealed after postoperative 3 months.

**Table 3:** Postoperative posterior segment complications and interventions after Ahmed valve implantation

Posterior segment complication	No. of eyes	Percentage of eyes	Procedure performed (No. of eyes)	Median time to onset of complication (Month)	Range (Months)
Choroidal detachment	44	17.7%	–	0.1	0–0.3
Ocular decompression retinopathy	8	3.2%	–	0.1	0–0.3
Hypotonic maculopathy	7	2.8%	–	0.8	0.1–2.5
Suprachoroidal hemorrhage	3	1.2%	Vitrectomy (1)	0.2	0–0.5
Endophthalmitis	3	1.2%	Vitrectomy (3)	11.3	0.1–23.7
Retinal detachment	3	1.2%	Vitrectomy (2)	3.2	1.6–8.8
Vitreous hemorrhage	5	2.0%	Vitrectomy (1)	1.2	0.1–3.2
Epiretinal membrane	2	0.8%	–	22.4	12.2–32.6
Cystoid macular edema	2	0.8%	Intravitreal bevacizumab or triamcinolone injection (2)	2.6	1.3–3.9
Proliferative vitreoretinopathy	1	0.4%	Vitrectomy (1)	10.3	10.3–10.3

Choroidal detachment occurred in the first postoperative week mostly and was resolved with observation. Vision was improved after resolution. Ocular decompression retinopathy was resolved in around 2 to 12 weeks. A central scotoma was noted while most of the cases were asymptomatic. No intervention was needed. Hypotonic maculopathy was treated with contact lens tamponade, pressure patching, or wound revision and resuturing.

Early infection occurred within the first month of the surgery, and late cases occurred years after the surgery and were because of the tube exposure. A patient underwent immediate vitrectomy and removal of the implant with vitreous sampling for culture, intravitreal vancomycin and ceftazidime injection. They were managed by intensive topical and systemic antibiotics. The culture of scleral patch of one patient was positive for staphylococcus species. In other patients, cultures grew streptococcus pneumonia and extensive retinal necrosis was the main operative finding.

High preoperative IOP, high myopia, anticoagulation, and systemic hypertension were attributed in most cases of suprachoroidal hemorrhage. Two patients had coughing after AVI and a small suprachoroidal hemorrhage was documented. It was confined to 1 or 2 quadrants so they were treated with conservative management with systemic steroids. One patient had a 360° appositional suprachoroidal hemorrhage after severe coughing that was documented on postoperative day 11 and drainage was performed with vitrectomy after 10 days following the liquefaction of suprachoroidal hemorrhage.

One serous retinal detachment didn’t require intervention. The other two eyes showed rhegmatogenous retinal detachment and required vitrectomy. The retina was attached after vitrectomy.

Four eyes with isolated vitreous hemorrhages were spontaneously resolved and one case underwent vitrectomy because vitreous hemorrhage compromised the shunt outflow resulting in increased IOP. In three patients, the tube was occluded by clot and hyphema from postoperative days 1 to 6. The eyes with cystoid macular edema were treated with intravitreal bevacizumab or triamcinolone and multiple injections were needed in one eye. The patient with proliferative vitreoretinopathy underwent vitrectomy but, resulted in phthisis bulbi, maintained VA to light perception.

## Discussion

Glaucoma drainage implants are shown to be successful in IOP reduction. When target IOP is not achievable with medical therapy or laser, glaucoma drainage implants should be chosen especially in secondary glaucoma in spite of the risk of complication because glaucoma ends up in irreversible blindness.

Although IOP can be successfully managed after AVI, posterior segment complications may develop. It is significant to understand the possible postoperative complications of AVI to maximize surgical efficacy. However, posterior segment complications of AVI have not been described in detail previously. In previous studies, rates of retinal complications were from 14 to 50% for the Molteno implant, 35% for the Krupin-Denver valve, 38–40% for the Krupin valve with a disc, and 22–48% for the Baerveldt implant [7–9]. Comparing these studies is hard due to different follow-up periods, different patient populations, small sample size, different surgical techniques, and different criteria for diagnosis. Our study involved a large sample size of eyes and the incidence of posterior segment complications of AVI was 31.4%.

The TVT study compared the efficacy of glaucoma drainage implant to trabeculectomy [4, 5]. The rate of postoperative complications was 34% during the first year which was similar to our study. The cumulative probability of failure was 29.8% in the tube group at 5 years [4, 5]. However since the Baerveldt implant was used in the TVT study, it’s difficult to accurately compare the results with ours. In the Ahmed Baerveldt Comparison Study, hyphema was the most common complication, accounting for 79% of intraoperative complications and no patient suffered an intraoperative suprachoroidal hemorrhage [10]. In the Ahmed Versus Baerveldt study, the complication rate was 52% in the Ahmed group which was higher than our results. The choroidal effusions accounted for 13%, which is similar to our study [11, 12].

Choroidal detachment, which also accounted for large proportion of posterior segment complication in our study, the incidence was 17.7%. In the TVT study, the incidence of choroidal effusion was 14% in tube which was similar to our results [5]. Another study reported 10% of choroidal effusion in Baerveldt valves and 15% in Ahmed valves [13]. The reported incidence of hypotonic maculopathy is 1.3–20% after glaucoma surgery [14]. We showed 2.0% of postoperative vitreous hemorrhage after AVI. Vitreous hemorrhage might have been a complication of the AVI, although contribution of underlying conditions could not be perfectly ruled out. Law et al. showed 5% incidence of vitreous hemorrhage after aqueous shunt placement [15]. The hemorrhage may result from, deep sclera sutures, bleeding from posterior segment conditions like suprachoroidal hemorrhage, or extension of bleeding from shunt entrance wound and iris neovascularization.

The reported incidence of suprachoroidal hemorrhage is 0.6–1.5% after trabeculectomy and 0.5–8.3% after shunt procedures [16]. In our study, intraoperative and delayed suprachoroidal hemorrhage was recognized in 1.2% which was similar with previous studies. Risk factors are high myopia, severe postoperative hypotony, high preoperative IOP, pseudophakia, aphakia, pulmonary disease, ischemic heart disease, systemic hypertension, and anticoagulation [17]. In our study, patients informed of their coughing prior to the onset of suprachoroidal hemorrhage. The visual prognosis was seriously bad, therefore, managing IOP, controlling risk factors, and monitoring of INR can decrease the risk for suprachoroidal hemorrhages after AVI.

The reported incidence of infectious disease after glaucoma surgery is 0.12–1.3% for endophthalmitis, 0.55–2.6% for blebitis [18, 19]. The endophthalmitis after AVI was reported to show more insidious onset than after cataract surgery and late-onset cases may develop after months to years due to infection through tube exposure [20]. Bacterial strains in endophthalmitis after AVI were reported to be more virulent, and showed high percentage of streptococcus species [21]. The incidence of retinal detachment was 1.2% in our study. Previous study revealed 5% incidence of retinal detachment after Molteno implant surgery, developing within 4 months following surgery [22].

Mermound et al. and Sidoti et al. found that preoperative VA worse than 20/200, diabetic retinopathy as cause of neovascularization, and young age were significantly associated with surgical failure for neovascular glaucoma in Molteno and Baerveldt shunt surgery [23, 24]. However, those authors did not separately assess IOP control and visual acuity in patients with posterior segment complications. In our study, older age, postoperative hypotony, and hypertension were found to be associated with posterior segment complications of AVI. Hypertension increases the fragility of vasculature and disrupts the integrity of the choroidal vasculature, that eventually lead to increased permeability [25, 26]. The structural vulnerability of the microvasculature, intraoperative or postoperative blood flow fluctuation may result in posterior segment complications in the old aged patients. The hypotony in postoperative period contributes to choroidal effusion and it causes a mechanical stress on the posterior ciliary artery which may result in suprachoroidal hemorrhage [27, 28]. Haga et al. showed that older age and postoperative hypotony were risk factors for choroidal detachment [29]. The results shown in our study are consistent with previous studies.

This limitation of our study is that the study was conducted by a retrospective design. However, we reported the most significant representation of postoperative posterior segment complications of AVI, both in regards to duration of follow-up and number of eyes. Our study quantified the long-term complication rates that can reshape surgical armamentarium and improve the quality of treatment of glaucoma.

## Conclusions

The overall incidence of posterior segment complications of AVI was 31.4%. Older age, hypertension, and postoperative hypotony were significantly associated with posterior segment complications of AVI.

## Data Availability

The datasets used and/or analysed during the current study are available from the corresponding author on reasonable request.

## Abbreviations

BCVA: Best corrected visual acuity
IOP: Intraocular pressure
LogMAR: Logarithm of the minimum angle of resolution
OCT: Optical coherence tomography
SD: Standard deviation
VA: Visual acuity
